# Hospital and Institutional News

**Published:** 1913-09-27

**Authors:** 


### HOSPITAL AND INSTITUTIONAL NEWS.

### THE DISCONTENT IN YORKSHIRE MENTAL HOSPITALS.

The Yorkshire papers have been full of correspondence concerning the unrest of mental hospital staffs in the West Riding. The complaints have been ventilated. What is the other side? For some time the Asylums Committee of the West Riding County Council has intended much to improve the conditions of service in the West Riding mental hospitals. The matter was, however, postponed owing to the proposed formation of the West Riding of Yorkshire Asylums Board. This Board has not yet been in existence for a year, but in July last it carried out the proposals of its predecessor in the control of the four large mental hospitals of the West Riding of Yorkshire in formulating and adopting new scales of salaries and wages for the whole of the staffs which are understood to compare well with the pay given elsewhere to mental hospital officials. Further, in one of the mental hospitals of the West Riding the hours of duty have already been reduced, particularly in the case of the nurses, and the scheme will shortly be completed. The visiting committees of the other three mental hospitals are at the present time considering schemes whereby a similar reduction in hours may be effected. The new scales of salaries have become known unofficially to the Secretary of the National Asylum Workers' Union, Mr. Gibson. Evidently the accompanying explanatory minute of the Asylums Board has been misunderstood or not sufficiently explicit, as Mr. Gibson, without waiting until the new scales came into force to find out exactly what was to be done, charges the Asylums Board in the public press with "grossly hoodwinking" and "playing the confidence trick" on its staff. We have reason to believe, however, that the cost of the increases in salary will for the present year alone amount to some thousands of pounds.

### IS A STRIKE OF MENTAL STAFFS POSSIBLE?

The threat of a strike can be seen in proportion when it is remembered that the Union hitherto has not gained official recognition. In any case a strike, even if it were really likely (which it is not), could not be a "down tools" in the accepted sense owing to the terms of service, which preclude any official from leaving without notice except at the sacrifice of a full month's wages and emoluments. Further, in the case of officials of over ten years' standing "a strike" would be a grave error, as they would sacrifice their right to the pension towards which they contribute annually. It is probably true to say that the National Asylum Workers' Union was determined to have its hand in the pie before the new scale of wages came into force and therefore found reason, in a misinterpretation of the minute, to suggest that the Board did not mean what it said.

### THE BRENTFORD INFIRMARY DISCLOSURES.

A discussion at a recent meeting of the Brentford Guardians reveals a very serious condition of things at the Brentford Poor-Law Infirmary. Colonel Carr-Calthrop, one of the members of the Board, asserted that many of the wards were overcrowded and contained more patients than were authorised by the Local Government Board regulations; that there were no proper sanitary conveniences in the separation wards; that the isolation accommodation was insufficient—lock cases having to be placed in the general wards and children suffering from measles being mixed up with those suffering from ringworm—and that there were no proper arrangements for warding severe surgical cases. In the course of a striking speech he said: "Things existed which were blots on their beautiful buildings, a nuisance to the nurses, and a horror to the medical staff, who knew only too well and feared the dangers which were thus invited. Some of the surroundings were such that he could not imagine how any medical man, not a regular surgical fanatic, would consent to perform any operation there that could possibly be postponed or avoided." The most astonishing thing in the discussion was that the truth of all these accusations was admitted without any qualification by the Chairman of the Infirmary Committee. He went a step further, indeed, and said: "The infirmary was a magnificent building, but was so absolutely lacking in the essentials for the treatment and care of the diseased that the diseases could not be satisfactorily dealt with, and instead of curing disease the conditions positively manufactured it owing to the lack of means of segregation." He asserted that the medical superintendent had reported upon these defects in 1906 and 1911, and that the matter had been brought before the Board on both occasions, but that no notice had been taken. Statements such as these leave one marvelling what kind of men and women the people of Brentford elect to be Guardians of the Poor.

### THE INADEQUACY OF LOCAL GOVERNMENT BOARD INSPECTION.

In considering the scandalous state of affairs revealed by this discussion the first question that arises is: How is it the Local Government Board has allowed such conditions to go on so long without protest? Under the General Consolidated Order the admission of a larger number of inmates to a ward than that fixed by the Local Government Board must be at once reported to it by the Clerk to the Guardians. Have these reports been sent, and, if so, why has the Local Government Board not taken steps to bring the Guardians to a sense of the duty they owe to the sick poor entrusted to them? The Chairman of the Guardians stated that medical inspectors from the Local Government Board had visited the infirmary and made no complaints. This raises the question as to the adequacy of the medical inspectorate of the Board. It must be remembered that there are only two medical inspectors for Poor-Law purposes for the whole of England and Wales. It is obviously impossible for two inspectors to make frequent and searching investigations into the adequacy of the medical arrangements made by Boards of Guardians over this large area, and one of the most urgent reforms required is a large increase in the number of medical inspectors. The fact that the medical superintendent's reports in 1906 and 1911 were ignored by the Guardians makes clear the importance of a suggestion we made in discussing the Draft Poor-Law Institutions Order. We pointed out that by the Order of April 4, 1868, the half-yearly reports of the medical officer of a Poor-Law institution had to be sent to the Poor-Law Board and that this requirement was rescinded by the Order of August 24, 1869, and we drew attention to the desirability of again making compulsory the forwarding of these reports to the Local Government Board. If such an enactment had been in force one cannot believe that the Brentford Board of Guardians would have been allowed to neglect so long one of the most important of their duties. At any rate, now that the matter has been raised so publicly, we may hope that drastic reforms will be carried out without any delay.

### THE FIVE ROYAL HOSPITALS.

The governors of the five Royal hospitals, St. Bartholomew's, Christ's, St. Thomas's, Bridewell, and Bethlem, and the Lord Mayor, aldermen, and sheriffs went this week as customary to service at Christ Church, Newgate Street. Of course, the most picturesque element of the occasion is the procession of the Christ's Hospital boys to the Mansion House to receive their annual gifts in coin and kind. The service, which was choral, was conducted by the Rev. T. R. Hine Haycock, the vicar, and the Rev. H. F. Westlake, minor canon and custodian of Westminster Abbey, preached the sermon.

### PROFESSIONAL SECRECY AT SEA.

Our recent article on "The Log of the Ship's Surgeon" has provoked enough quotation and comment for us again to drive the following points home. The surgeon's log is a professional document, to be read by professional men alone; a private case-book which is, or should be, sacred to all but the three individuals primarily responsible for the well-being of the ship's community. As such it should be clear and authoritative, a true record which may be produced as evidence, as the captain's log-book is admissible as evidence. When it is degraded into a record to be read by clerks and compradors, it is the business of the ship's surgeon, in his own interests, to make it as little informative as possible. Then, of course, it ceases to be a log-book; it becomes a charivari from which the office clerks may perhaps extract some amusement, but which no Court will admit as evidence. Its value to a ship's company will consequently be *nil*. And that is exactly what it should be if a company persists in requiring its surgeons to violate one of the few conventions that still enable medical practitioners to claim that they are members not of a trade but of a profession.

### LAST OF THE FARMFIELD REFORMATORY.

The Farmfield Reformatory, established for the reception of female inebriates, is to close at the end of the year. This institution was opened some ten years ago, when the Government empowered local authorities to take steps for the treatment of habitual drunkards. This movement came about owing to the eccentricities of the notorious Jane Cakebread, whose periodical appearances at the police courts for drunkenness came to be a public scandal. The London County Council decided to make use of the Farmfield institution for female drunkards committed from the police courts, and it also arranged to board out others in provincial institutions. After ten years the Council has arrived at the conclusion that women who degenerate into habitual drunkards cannot be cured. Of six hundred cases passed through Farmfield not more than 18 per cent. were reported to be doing well subsequently, but even these were doubtful, for drunkards have a habit of assuming other names when charged. So the Council is abandoning its efforts to reclaim drunkards. At Farmfield there are about a hundred women undergoing the treatment; about three hundred others are boarded out in the country, and they too will be discharged at the end of the year. The efforts of the London County Council to reform these women cost the ratepayers ten thousand a year in spite of the grants received from the Government, and it is now thought that results do not justify the continuance of this expenditure. Of course, there is still a possibility that the Government may yet decide to increase its grant and so entice the London County Council to continue. Otherwise, at Christmas, some four hundred female drunkards will be freed. As to the future, what will happen is that inebriates coming before the magistrates will be sent to prison. Of the women now in the institutions many of them have recorded against them convictions ranging from 30 to 150, and so it must be admitted that they really seem incorrigible.

### WORKERS' HOSPITAL FUND AT CAMBRIDGE.

We publish elsewhere an account of the annual meeting of this Fund. The past year for the Workers' Fund has been one of great activity and stress of work, but, despite the very strenuous efforts which have been made on all sides in common with other organisations of similar character, the Workers' Fund has suffered in consequence of the passing into operation of the National Insurance Act, but it is hoped that the workers who already contribute to the Fund and those who have not as yet given the Fund their support, however small, will realise that there is as great a need now, if not greater, for the hospital with all the advantages which it affords. The year now referred to commenced with 112 centres, and the number has dropped to 94, but against those centres which have for various reasons ceased to contribute the centre which has been established at Messrs. Chivers and Sons, Histon, has to be set-off. The total amount raised by the Fund for the year to June 30, 1913, was £555, and the expenditure for the year amounted to about £17 1s. 2d. The Convalescent Fund has again proved of the utmost value.

### THE FORBES WINSLOW MEMORIAL.

Owing to the death of Dr. Forbes Winslow, the British Hospital for Mental Disorders and Nervous Diseases is under the management of a new lay executive committee, and is being entirely re-organised. An increased medical staff has been appointed, and as soon as the lease expires new premises will be found. We understand that there have been on an average 3,000 borderland cases treated annually, and it is hoped that the usefulness of this institution will be considerably increased when its reorganisation is completed.

### MEDICAL FRIENDS

From time to time it happens that a young medical man obtains little distinctions: he wins a class medal, or gets a paper printed in one of the medical journals, or has a present made him by a grateful patient. Then, if his evil star is in the ascendant, kind friends get thinking about the local press; the latter responds willingly enough, and a little bit of harm to the medical profession's name is the result. Such a thing happened—we repeat, it is always happening—the other day. The young doctor's name does not matter, nor those of the papers; the local paper was published near London. The subject of the article might, however, be mentioned; it was that of nascent iodine in the treatment of consumption. Now nascent iodine, liberated at the site of the lesion by means of hydrogen-peroxide solution acting on sodium iodide (administered orally) from the blood stream of the part affected, has had some sustainedly good reports, in the case of intra-nasal lupus, from the rhinological clinic at the Finsen Institute at Copenhagen and other centres. It was, therefore, a good idea of another medical man, Dr. Curle, of Glasgow, some time ago to try the method, or some approximation to it, in consumption. We confine ourselves to *a priori* considerations, because in the little paper under notice there is nothing in the way of appraisement of results except the vaguest clinical estimates. How many thousands upon thousands of "cures" for consumption, well forgotten now, have not in the past received just such recommendation? In sum, an achievement not yet (as the author would no doubt admit) worth the setting of half a column of type in a lay journal.

### DUST AND ENTERITIS IN SOUTHWARK INFIRMARY.

Nine children have now died in Southwark Infirmary from enteritis, and there are still thirty-five cases which are due, in the opinion of Dr. Bruce, the medical superintendent, to dust poisoning, probably arising from the refuse heap near by that is owned by the Camberwell Borough Council. The Local Government Board, whose inspector visited the siding, in a letter to the Camberwell Borough Council, state that the inspector reported that the arrangements for dealing with the refuse were unsatisfactory and at times gave rise to a nuisance. Having regard to the close proximity of the infirmary and other institutions, the Board suggested that the Council should discontinue the use of this siding for the transhipment of refuse from the carts to railway trucks. The suggestion is a distinct triumph for the Southwark Guardians.

### PROPOSED ACTION BY THE COUNCIL.

The letter of the Local Government Board has been considered by the works committee of the Camberwell Borough Council, who report that they have again surveyed the site and have given instructions for every possible care to be taken in the handling of the refuse, and were requesting the Railway Company to ensure that all loaded trucks were removed every night as usual. The partly filled trucks will remain at the siding, as usual, and the remains of the dust, coming in late at night, which cannot be dealt with, will, we presume, be left on the tip till the following morning. Herein lies the danger to the public health from a mass of rubbish, already left in the dust-bins for a week, being further left all night uncovered. The public health committee ask the Council to request the Local Government Board to hold an inquiry. They have considered and endorsed a report of Dr. Stevens, the medical officer of health, that the deaths in the infirmary are not due to the refuse. It is suspect still.

### PARENTS PUNISHED FOR REFUSING TO ISOLATE.

For refusing to allow their children when suffering from infectious disease to be removed to hospital, notwithstanding the grant of an order for such removal by a justice of the peace—as was reported in our columns three weeks ago—two Greenford parents were summoned last week at Brentford Petty Sessions and mulcted in penalties of forty shillings. The evidence went to show that defendants wilfully defied the medical officer of health for the district, Dr. Hope, and also subjected to rough treatment a nurse who called upon them in connection with the matter. The defence set up was to the effect that the children were properly isolated at home, and Dr. Cummings, the medical man in attendance, agreed that isolation was properly carried out, although by no means ideal. In giving the decision of the Bench, the Chairman said defendants had no right to obstruct an order made for the benefit of the public health. The magistrate's order had been made on the certificate of the medical officer, who considered the children should be removed, and for defendants to put their opinion against that of an expert was ridiculous.

### SCHOOL CLINIC POSTS AND THEIR SALARIES.

The recent appointment of Dr. D. Leighton Davies to the post of ophthalmic surgeon in connection with the Cardiff school clinic was the occasion of an instructive discussion on the salaries offered and the work demanded of medical men engaged in school work. Dr. Davies offered himself conditionally on the work being limited to one morning of two hours a week, on a different arrangement being come to in six months' time, and at a salary of £50 a year. He stated that the salary was inadequate, hence the conditions which he had laid down. The medical officer of health, Dr. Walford, explained that eye work was the most important section of the school clinic, an explanation which will be endorsed by all who read the expert description of what it entails which we publish on another page of this issue. Other speakers also insisted on the fact that for special work the demand for more than £50 per annum was not extravagant. The truth, of course, is that in order to get any high value from a school clinic it is absolutely imperative that men specially qualified should be appointed to these posts. This can only be done by offering salaries adequate enough to tempt the best skill available. Here, as elsewhere, the right, and in the long run the economical, policy is to offer first-rate salaries and then to insist on first-rate qualifications.

### RADIUM TREATMENT FOR POOR-LAW PATIENTS.

The possibility of establishing in London a Radium Department for the treatment of patients in Poor-Law infirmaries was discussed in our columns of August 30. At the last meeting of the Wandsworth Board of Guardians a resolution was carried asking the Local Government Board to place the department under the control of the Metropolitan Asylums Board. Mr. D. Grundy, who proposed the resolution, pointed out that there were numerous cases of poor patients in infirmaries unable to obtain admission to the special hospitals, who would, in all probability, be benefited, if not cured, by the treatment. Mr. P. Rees, the vice-chairman, in seconding, said that no single Board of Guardians could supply the necessary funds for the equipment of a Radium Department. At present the Metropolitan Asylums Board were treating patients suffering from ringworm with radium, and, consequently, it would be an easy matter for them to extend their department. The Board decided to ask the various Metropolitan Poor-Law authorities to support the proposal, the result of which will be awaited with interest.

### THE SICK PAY OF WORKHOUSE OFFICIALS.

The Chairman of the Finance Committee of the Scarborough Board of Guardians referred last week to the fact that one of the workhouse officials while off duty for reasons of health claimed full wages from the Guardians, while he had been receiving 10s. per week sick pay under the Insurance Act. In the view of his committee it was unreasonable that any official should receive through the operation of National Insurance more money when off duty than when he was well and at work. The Chairman also pointed out that this was the Local Government Board's opinion, since the Board had stated it clearly in a communication to the Northallerton Guardians, one of whose officials had made a similar claim. It was finally decided to adopt a recommendation of the committee by which the Guardians should enter into an agreement with their officials to deduct the amount of sick pay from their salaries. When an unexpected course of events allows one party to a previous agreement to a benefit which was not contemplated at the time that the agreement was drawn, it is only human nature that the favoured of fortune should try to make the most of his good luck. At the earliest opportunity the other party can rightly, of course, draft new agreements to meet the change of circumstances that has intervened.

### THE LATEST FORM OF HOSPITAL ENTERPRISE.

At the last Ideal Home Exhibition at Olympia Prince Alexander of Teck, on behalf of the Middlesex Hospital, secured the sympathies of many well-known ladies to decorate a number of dinner tables according to their individual tastes. The whole exhibit was so great a financial success that for this year's exhibition Prince Alexander has initiated a scheme for the decoration of a "perfect" home, each room of which has been arranged, decorated, and furnished by a well-known lady. Queen Alexandra, Princess Alexander of Teck, and other eight ladies have promised to take part. The names of these ladies with the rooms they are undertaking are as follows: Queen Alexandra, a day and night nursery; Princess Alexander of Teck, an Adams drawing-room; the Duchess of Rutland, a Georgian dining-room; the Marchioness of Anglesey, an English eighteenth-century bedroom; the Countess of Lytton, an early sixteenth-century Venetian bedroom; the Countess of Plymouth, a William and Mary library; Lady Islington, a seventeenth-century parlour; Lady Halford, the hall; Lady Speyer, a Queen Anne boudoir; Viscountess Wolseley, an old English garden. We are informed that the plans and scheme of decorations are almost complete, and that the furniture either has been chosen or is being specially made. The nine rooms and the garden will occupy the whole of the Minor Hall in the Gallery at Olympia. The exhibition opens on October 9, when we hope to publish an account of its chief features. The Middlesex Hospital has gained already a great reputation for inventing novel entertainments likely to attract the support of wealthy people, and this latest attempt will be watched with interest.

### OUTDOOR WORK FOR MENTAL PATIENTS.

At the Montrose Royal Mental Hospital the Commissioner in Lunacy, Dr. John McPherson, has recently noted some interesting figures concerning the employment of patients, and particularly the large proportion for whom outdoor work has been found. Of the 689 patients on the register 420 are stated to be industrially employed, half of whom, for 204 may be considered half, are working in the grounds and gardens. The work which the women patients accomplish, it is interesting to note, since outdoor employment is not always easy to find for women in mental hospitals, consists mainly of fruit picking, but it is hoped that other forms of congenial work less dependent on the season may be found in time. It is a well-known fact that patients suffering from mental disease, who are not too ill to be employed at all, make in certain respects even better workers than their more healthy fellows. They are more careful, more concentrated, less inclined to "play," and, though as a rule their work is slowly accomplished, it is generally very thorough and painstaking when the right sort of supervision is found.

### A DISTINGUISHED MENTAL SUPERINTENDENT.

The recent retirement of Dr. Alexander Reid Urquhart from the post of physician-superintendent of the James Murray Royal Mental Hospital, Perth, removes from the active list not merely a distinguished administrator, but a former medical editor of distinction. Appointed to the post which he has just resigned in 1879, Dr. Urquhart has had a long spell of administrative responsibility, but his life has been a varied one in many ways. He has held appointments both in England and Scotland, he has travelled considerably, and he has edited the *Journal of Mental Science*; while in other spheres he has been active, having been a participator in many public affairs in Perth. His papers on "Asylum Construction," "Classification of the Literature of Insanity," and on asylum organisation and administration form part of the literature of his subject. In the last report for which he is responsible Dr. Urquhart had some interesting utterances on the relation of insanity to drunkenness, his experience being that drunkenness has been the effect rather than the cause, and also on the relation of insanity to longevity. In this case, in spite of the average age at death in the Murray institution being seventy-three, Dr. Urquhart inclines to the view that generally insanity is unfavourable to prolonged life. Readers of The Hospital will recall a long letter which Dr. Urquhart sent us some time ago on the hours of work in mental hospitals.

### A NEW DEPARTMENT OF ARCHITECTURE.

Since the passing of the Mental Deficiency Act Boards of Guardians throughout the country have been considering the best way in which colonies for the feeble-minded may be started. As there are, comparatively speaking, few of these in existence, visits have been hastily paid to those available. The other day, for instance, the committee of the Bradford Board of Guardians, who have supervision of the recently started Bowling Park Colony, sent a deputation to inspect the industrial colony at Darenth, which, by the way, has been a sort of Mecca for such pilgrimages of late. The Bradford committee has adopted the deputation's report, which recommends detailed pavilions accommodating from forty to forty-five beds for the residential buildings, which are also to contain an observation room for the attendant and a day room. The buildings are to be generally of the bungalow type. There is to be an administrative block, with a central kitchen and quarters for the resident staff, four pavilions for different classes of patients, and these are to be proceeded with at once. Surplus accommodation is also to be provided. It is also held that the larger the institution the more satisfactorily can it be administered. We mention these points, simple and summary though they are, since all, or practically all, the existing colonies have been adaptations, like the Darenth institution, for instance, and therefore the architectural type has yet to be worked out. When we remember what muddles have been caused in hospital buildings from the fact that architects so long refused to realise that hospital architecture was a special department needing special training and study we may well remind the profession here of a new department of hospital architecture which is likely to be much in request during the next few years, and to which, therefore, architects should devote study and attention.

### MENTAL STAFFS AND PREVIOUS TREATMENT.

At an inquest held last week on a mental patient who died within twenty-four hours of admission to the Brighton County Borough Mental Hospital at Hayward's Heath the Coroner made some comments on the way in which patients were sent to mental hospitals without any information as to their previous treatment. This, he said, was the weak spot in the system. The inquest was held because the patient, whose violence had led to her being sent to the institution, was on her arrival under the influence of a drug which was not known to the mental hospital's doctors. Dr. Ambrose Emerson, of Eastbourne, who had attended the deceased before her removal to the institution, in giving evidence, said that a relieving officer should be provided with a form which medical men could fill up. Mr. Charles Planck, medical superintendent of the Borough Mental Hospital, said he was present at the *post-mortem* examination and did not think either the removal or the administration of drugs had anything to do with the patient's death, which he attributed to acute inflammation of the kidneys. Mr. H. J. Foster, assistant medical officer of the institution, said that on her arrival he obtained information concerning the history of the case from the relieving officer as the patient was apparently under the influence of a drug. It is clear that in these cases of unexpected admission some ready means should be available for the institutional staff to learn the previous history of the case.

### THE LONDON FEVER HOSPITAL.

Attention has often been drawn, both in these columns and elsewhere, to the undoubted fact that in Great Britain the wealthy and the poor can, and do, command better facilities for treatment during serious illness than the less affluent of the middle classes, who cannot afford to pay the high fees of nursing homes and specialists, yet are ineligible for charitable institutions. One of the few London hospitals which help to remedy this unsatisfactory state of affairs is the London Fever Hospital, Islington, which exists primarily for the reception of scarlet fever cases in the middle classes, and has for many years done most valuable work. It should be added that typhoid fever, diphtheria, measles, and German measles are also accepted. Unfortunately the very moderate fees charged do not cover the cost of maintaining the hospital, which has to solicit the benefactions of the public. Unfortunately, too, the latter do not at present suffice to make ends meet. The Secretary, Major Christie, is, in consequence, issuing a personal appeal for more subscriptions, of which he hopes to secure a thousand of one guinea each. In an excellent letter he emphasises subscription as a species of insurance, for such it really is, owing to the privileges which are afforded to subscribers, their families, servants and dependants in general. It gives us pleasure to afford this appeal such further publicity and recommendation as is in our power, for we know the excellent work which this hospital carries on, and the difficulties under which the management strive to keep it going in uncurtailed usefulness.

### HOSPITAL SECRETARY'S SUDDEN ILLNESS.

We are sorry to learn that Mr. J. A. Rudd, the secretary of the Coventry and Warwickshire Hospital, has been taken suddenly ill, and had to be carried at once to the hospital, where an operation was performed. Till Mr. Rudd is able to undertake his duties again, which will not be for several weeks, Mr. Barnard Franklin has been appointed temporary secretary.

### THE MILNE SYSTEM AND MEDICAL PREROGATIVE

What is there in the Milne system for the treatment of scarlet fever which causes administrative trouble in so many cases where it is advocated? It will be remembered that in January 1912 Dr. Butchart, then medical superintendent of the Blawarthill Hospital, under the Renfrew and Clydebank Joint Hospital Board, was actually dismissed because he refused to abandon the existing method in favour of the Milne system as a matter of routine. It is true that the inhabitants of Clydebank were so much disgusted at this attempt to impose treatment by committee on the neighbourhood that a public presentation was quickly made to him, and thereby the right of medical men not to be dictated to in the matter of treatment was vindicated. The Milne treatment, however, has again caused trouble. As a result of a scarlet fever epidemic the Arbroath Town Council passed a resolution last week to the effect that "a nurse or nurses be employed by the local authority with a view to the adoption of the domiciliary treatment of scarlet fever after the Milne method where desired." At a subsequent meeting of the Public Health Committee, Dr. Yule refused to be dictated to in medical matters by the Town Council; as a result the latter caused a special meeting to be called. Domiciliary treatment, said Dr. Yule at this meeting, as the hospital is full, must be left to general practitioners, and he refused to have any part in imposing the Milne system upon them. Bailie Anderson and Mr. Mackenzie then moved and seconded a motion, which was adopted, that the town clerk should circularise the medical profession with an intimation that the local authority had resolved to appoint nurses for the domiciliary treatment of fever cases under the Milne system. A further motion was carried requesting the Local Government Board to approve what had been done! Dr. Butchart stood firm and carried his point. Dr. Yule, we hope, will do the same.

### THE BANE OF INFLUENCE IN PHTHISIS WORK

Any system of administration is liable, and all suffer from, the baneful effects of influence in the selection of second or third best candidates for responsible posts. But it is noteworthy that influence is usually most virulent when a new set of appointments comes into vogue. This is very strikingly shown, we fear, in the recently created tuberculosis service. A case has been reported to us in which a tuberculosis officer was appointed who had never seen the pneumothorax treatment, was quite beaten by a throat to see, could not read German, and knew next to nothing of the literature of tuberculosis. Why, then, was the appointment? The most likely reason appears to be that he was the son of a local tradesman, and had a local qualification. What is the reverse of the picture? We can quote another case in which a qualified medical man, who has specialised in tuberculosis, who has had five years' sole charge of a sanatorium, knows German, has made a study of the various special treatments now available, and is unusually well read for an Englishman in the foreign literature of the subject, was not even on the short list for a post which was given to a candidate of the same type as that first described. In another case sanatorium experience was stipulated for in the advertisement, and the successful man had none! We believe these instances to be far from exceptional, and shall be glad to receive further evidence for, or against, the contention that tuberculosis appointments are being grossly manipulated at the present time.

### THIS WEEK'S DRUG MARKET.

Business in the Drug market has been on an unimportant scale during the week, and price changes have been of minor importance. Makers of salicylic acid and salicylates have announced a small reduction in price, which is probably due to competition. Very high prices are still asked for Russian cantharides. Belgian chamomiles are rather lower in price. Practically no business is being done in cod-liver oil, and prices are practically unchanged. Ergot of rye is, if anything, rather lower. Opium is quiet, but prices are well maintained, and it is thought in some quarters that any further reduction would not be substantial. American peppermint oil is dearer. A further advance in the price of santonin would not come as a surprise in spite of the extreme figure now quoted. At the recent public sale of drugs in Mincing Lane menthol sold at lower prices; Cape aloes and cardamoms were rather dearer; ergot of rye, Jamaica honey, and ipecacuanha were slightly lower.

